# Clinical Relevance of Elevated Soluble ST2, HSP27 and 20S Proteasome at Hospital Admission in Patients with COVID-19

**DOI:** 10.3390/biology10111186

**Published:** 2021-11-15

**Authors:** Ralph Wendt, Marie-Therese Lingitz, Maria Laggner, Michael Mildner, Denise Traxler, Alexandra Graf, Pavla Krotka, Bernhard Moser, Konrad Hoetzenecker, Sven Kalbitz, Christoph Lübbert, Joachim Beige, Hendrik Jan Ankersmit

**Affiliations:** 1 Department of Infectious Diseases, Tropical Medicine, Nephrology and Rheumatology, St. Georg Hospital, Delitzscher Str. 141, 04129 Leipzig, Germany; Ralph.Wendt@SanktGeorg.DE (R.W.); sven.kalbitz@sanktgeorg.de (S.K.); christoph.luebbert@sanktgeorg.de (C.L.); Joachim.Beige@sanktgeorg.de (J.B.); 2 Laboratory for Cardiac and Thoracic Diagnosis, Regeneration and Applied Immunology, Medical University of Vienna, Research Laboratories Vienna General Hospital, Waehringer Guertel 18-20, 1090 Vienna, Austria; marie-therese.lingitz@meduniwien.ac.at (M.-T.L.); maria.laggner@meduniwien.ac.at (M.L.); michael.mildner@meduniwien.ac.at (M.M.); denise.traxlerweidenauer@gmail.com (D.T.); bernhard.moser@meduniwien.ac.at (B.M.); 3 Department of Anaesthesia, Intensive Care Medicine and Pain Medicine, Medical University of Vienna, Waehringer Guertel 18-20, 1090 Vienna, Austria; 4 Department of Dermatology, Medical University of Vienna, Waehringer Guertel 18-20, 1090 Vienna, Austria; 5 Division of Cardiology, Department of Internal Medicine II, Medical University of Vienna, Waehringer Guertel 18-20, 1090 Vienna, Austria; 6 Center for Medical Statistics, Informatics and Intelligent Systems, Medical University of Vienna, Spitalg. 23, 1090 Vienna, Austria; alexandra.graf@meduniwien.ac.at (A.G.); pavla.krotka@meduniwien.ac.at (P.K.); 7 Division of Thoracic Surgery, Department of Surgery, Medical University of Vienna, Waehringer Guertel 18-20, 1090 Vienna, Austria; konrad.hoetzenecker@meduniwien.ac.at; 8 Division of Infectious Diseases and Tropical Medicine, Department of Internal Medicine II, Leipzig University Medical Center, Liebigstr. 20, 04103 Leipzig, Germany; 9 Department of Internal Medicine II, Martin-Luther-University Halle-Wittenberg, 06108 Halle/Saale, Germany

**Keywords:** COVID-19, HSP27, sST2, ARDS, biomarker, 20S proteasome

## Abstract

**Simple Summary:**

Since the outbreak of the Coronavirus Disease (COVID-19) disease in 2019 immunological research is continuing to debunk contingent pathomechanistic interpretations. Currently the COVID-2019 infection is described in humans as a state of hyperinflammation. As the COVID-2019 pandemic continued we collected serum (first blood draw) from patients that were admitted to the hospital (2020/21). Here we show that proteins that indicate “immune decay” (heat shock protein 27 and 20S proteasome) and immune suppressive soluble suppression of tumorigenicity 2 (sST2) were massively increased in those COVID-19 patients. In addition, we demonstrated that those proteins were associated with mortality, invasive ventilation, and oxygen support. Facit: Our results indicate that COVID-19 disease can also be construed as disease that causes an immunological disintegration. Historically it is notable to report that sepsis pathophysiology underwent a similar metamorphosis. Until 2003 the clinical picture of sepsis was seen as a consequence of a hyperactive immune system. This scientific interpretation tottered, and clinical research has evidenced that septic patients are in a state of immunologic default. Based on our serological data we believe that the concept of “COVID-19 induced hyperinflammation” is ready to undergo a critical appraisal and calls for cytoprotective therapeutic interventions.

**Abstract:**

Although, severe acute respiratory syndrome coronavirus-2 (SARS-CoV-2) represents one of the biggest challenges in the world today, the exact immunopathogenic mechanism that leads to severe or critical Coronavirus Disease 2019 (COVID-19) has remained incompletely understood. Several studies have indicated that high systemic plasma levels of inflammatory cytokines result in the so-called “cytokine storm”, with subsequent development of microthrombosis, disseminated intravascular coagulation, and multiorgan-failure. Therefore, we reasoned those elevated inflammatory molecules might act as prognostic factors. Here, we analyzed 245 serum samples of patients with COVID-19, collected at hospital admission. We assessed the levels of heat shock protein 27 (HSP27), soluble suppressor of tumorigenicity-2 (sST2) and 20S proteasome at hospital admission and explored their associations with overall-, 30-, 60-, 90-day- and in-hospital mortality. Moreover, we investigated their association with the risk of ventilation. We demonstrated that increased serum sST2 was uni- and multivariably associated with all endpoints. Furthermore, we also identified 20S proteasome as independent prognostic factor for in-hospital mortality (sST2, AUC = 0.73; HSP27, AUC = 0.59; 20S proteasome = 0.67). Elevated sST2, HSP27, and 20S proteasome levels at hospital admission were univariably associated with higher risk of invasive ventilation (OR = 1.8; *p* < 0.001; OR = 1.1; *p* = 0.04; OR = 1.03, *p* = 0.03, respectively). These findings could help to identify high-risk patients early in the course of COVID-19.

## 1. Introduction

The severe acute respiratory syndrome coronavirus type 2 (SARS-CoV-2) pandemic is one of the largest challenges the world faces today. It remains difficult to estimate the frequencies of severe disease courses and fatalities linked to Coronavirus Disease 2019 (COVID-19), due to the number of undiagnosed and asymptomatic infections, which varies with differences in susceptibility and vulnerability among different populations. The reported hospitalization rates for patients with diagnosed SARS-CoV-2 infections varies between 7 and 25% [1,2]. The Robert Koch Institute reported 23% mortality among patients hospitalized with COVID-19 in Germany [2]. Among patients that required intensive care in Germany, mortality rates were 30% and 36% for patients that received ventilation support [2]. Clinical deterioration or the development of severe or critical disease typically occurs later in the disease course, at least 7 to 10 days after the infection. Although several immunopathological mechanisms that lead to severe COVID-19 have been discovered, the exact immunopathogenesis remains incompletely understood. Several studies have indicated that pulmonary inflammation in COVID-19 was associated with high systemic plasma levels of inflammatory cytokines, which resulted in the so-called “cytokine storm” [3,4,5,6]. Inflammatory cytokines are released by infected cells, alveolar macrophages, recruited T lymphocytes, monocytes, and neutrophils. They increase the permeability of lung epithelium and reduce its barrier function. As a result, pulmonary edema fills the alveolar spaces, followed by the formation of hyaline membranes, similar to the early-phase features of acute respiratory distress syndrome (ARDS) [7,8]. Indeed, cytokine overproduction is a characteristic feature of ARDS; consequently, several studies have suggested that a cytokine storm may be the main pathogenic factor in COVID-19-related ARDS. [9,10]. Additionally, the overproduction of inflammatory molecules is involved in the subsequent development of microthrombosis, disseminated intravascular coagulation, and multiorgan-failure. This imbalance between pro- and anti-coagulants was commonly found in patients that died of COVID-19, along with elevated d-dimer concentrations [9,11,12].

Indeed, COVID-19-associated ARDS displays several general characteristics of non-COVID-19-associated ARDS, such as impaired gas exchange and characteristic computed tomography (CT) findings. However, COVID-19 associated ARDS exhibits a more variable clinical appearance than non-COVID-19-ARDS [7,13,14]. Previous studies have also shown that reduced numbers of CD4+ and CD8+ T-cells were associated with severe COVID-19. That finding indicated that COVID-19 induced an immunodeficient state, comparable to the sepsis-induced immune-cell apoptosis observed in non-COVID-19-related ARDS and bacterial sepsis [3,4,8,15,16,17].

Previous studies investigated immune-cell apoptosis in patients with sepsis [18]. They demonstrated that this phenomenon was associated with increased levels of soluble forms of circulating cluster of differentiation 95 (CD95), tumor necrosis factor 1 (TNF1), and interleukin-1-converting enzyme. Those findings suggested that lymphopenia, Th2 predominance, and subsequent immunodysfunction were caused by an aberrant state of T-cell activation that involved the CD95 pathway, the shedding of death-inducing receptors, and a tendency for CD4+ T-cells to undergo activation-induced cell death. Further studies [19,20] implicated the involvement of elevated levels of 20S proteasome and caspase cleaved cytokeratin 18 (cCK18) in immune-cell apoptosis. 20S proteasome is a multi-catalytic proteinase complex involved in the enzymatic degradation of ubiquitinated proteins, and it serves as a marker of cell damage and immunological activity in autoimmune diseases. CCK18 is a marker of apoptosis that is not detectible in vital or necrotic cells, which indicates that cCK18 is associated with a higher apoptotic turnover during sepsis. In support of the hypothesis that, in sepsis, Th2 predominance arises from a shift from Th1 to Th2-biased cell proliferation, Brunner et al. [21] showed an increase in the levels of soluble suppressor of tumorigenicity (sST2), a member of the interleukin-33 (IL-33) family. Indeed, sST2 is predominantly expressed in type-2 T helper (Th2) cells, but not Th1 cells, which suggests that sST2 plays a role in cell proliferation and the promotion of the Th2 immune response. Additionally, Mildner et al. [22] showed that sST2 was mainly produced in alveolar epithelial cells in the lung and cardiac myocytes. They also detected a strong augmentation of sST2 serum levels within 24 h post-LPS infusion, which implied a strong inflammatory response, accompanied by the secretion of inflammatory cytokines, such as IL-6 and TNF-α. Moreover, heat-shock-protein 27 (HSP27), a member of the small heat shock protein family, was identified as an early marker of pulmonary damage and disease severity in patients with chronic obstructive pulmonary disease (COPD) [23,24]. In a recently published study from Abers et al., sST2 was independently associated with higher mortality in patients suffering from COVID-19. They also identified elevated IL-6 at hospital admission as early predictor for increased mortality [25]. In another study from Chen et al. IL-6 besides inflammatory markers such as C-reactive protein (CRP), d-dimer and ferritin were associated with worse outcome [4]. Although, the above-mentioned inflammatory markers had been described as prognostic factors, in a meta-analysis from Leisman et al. [26] serum levels of CRP, IL-6 and ferritin were compared between severe COVID-19 and other inflammatory diseases such as Cytokine Release Syndrome (CRS) and ARDS, revealing markedly decreased secretion of these markers compared to CRS and sepsis.

Considering these observations, we hypothesized that concentrations of other inflammatory molecules and sST2 might be elevated in our patient cohort with severe COVID-19, and they might act as prognostic markers of survival. In the present study, we investigated the survival of 245 patients with COVID-19 and determined serum levels of sST2, TNF1 receptor (TNFR1), cCK18, HSP27, and 20S proteasome in specimens collected at hospital admission. We then explored which inflammatory markers were associated with a poorer prognosis in overall survival, 30-, 60- and 90-day survival and in-hospital mortality. In addition, we investigated which parameters could indicate higher risk for patients requiring ventilatory assistance or oxygen therapy.

## 2. Materials and Methods

### 2.1. Study Population

From August 2020 to February 2021, serum samples were collected from patients infected with SARS-CoV-2 that were treated in the Department of Infectious Diseases and Tropical Medicine at St. Georg Hospital, in Leipzig. The treating unit included a standard hospital ward (33 beds) and an intermediate-care unit with 7 beds. At the time of admission, routine laboratory parameters were determined such as complete blood counts. In addition, laboratory parameters and clinical information, such as comorbidities and the course of disease, were derived from the medical history of each patient. Among the 270 serum samples, 25 were excluded, due to duplicate samples and patients with missing values. Thus, 245 patient samples were included in this study.

None of the patients had been vaccinated for SARS-CoV-2 prior to study inclusion. Patients that required extracorporeal membrane oxygenation (ECMO) procedures were transferred to another hospital, which was considered a discharge in our analyses.

All patients provided written informed consent prior to blood collection and participation.

### 2.2. Serum Samples

Venous blood was drawn within 48 h of hospital admission. Whole blood was centrifuged at 4075× *g* for 4 min at room temperature. After centrifugation, aliquoted samples were stored at −80 °C, until tests were performed.

### 2.3. Quantification of Serum HSP27, TNFR1, sST2, cCK18, and 20S Proteasome

We assessed serum levels of HSP27, sST2, and TNFR1 with commercially available ELISA kits (R&D Systems, Minneapolis, MN, USA), as described previously assessed with a commercial ELISA kit (Enzo Life Sciences, Farmingdale, NY, USA). All ELISA assays were performed according to the manufacturers’ protocols. Colorimetric measurements were performed using Tecan F50 infinite microplate reader (Tecan Group, Männedorf, Switzerland) with Magellan software (version 7.2, Tecan, Männedorf, Switzerland). Analytes were quantified according to external standard curves.

### 2.4. Statistical Analysis

Statistical analyses were performed using R, version 3.6.2. Data on 245 patients positive for SARS-CoV-2 were analyzed and associations between inflammatory molecules (HSP27, sST2, TNFR1, CK18, 20S proteasome) and the following endpoints were investigated: Overall mortality, 30-, 60- and 90-day-mortality, in-hospital mortality, requirement of ventilatory assistance or oxygen therapy. For the analysis of overall mortality, uni- and multivariable Cox regression models were computed. Binary endpoints were analyzed using uni- and multivariable logistic regression as well as ROC curves. The results of the Cox regression models are presented using Hazard Ratios (HR) with corresponding 95% Confidence Intervalls (CI) and for the binary endpoints, results of the logistic regression models are presented as Odds Ratios (OR) with 95% CI. ROC curves and AUCs with 95% CIs are presented. All possible predictors were at first assessed in an univariable analysis. Subsequently, multivariable models were computed including only significant predictors from the univariable analyses. Median concentrations of assessed inflammatory markers were compared using Mann-Whitney-U-test. Due to the exploratory character of the study, no correction for multiplicity was performed and *p*-values < 0.05 were considered statistically significant.

## 3. Results

### 3.1. Study Population Characteristics

After excluding duplicate samples and patients with missing data, we included serum samples of 245 patients with SARS-CoV-2 infections. Demographic, clinicopathological, and laboratory details are shown in Table 1 and Table 2. Median values with interquartile range (IQR) of measured parameters for mortality endpoints are depicted in Table 3. Detailed description of the World Health Organization (WHO)—Outcome Classification for COVID-19 is depicted in the Supplementary Data (Table S1) [27].

The study population displayed a female to male ratio of 46.1:53.9 with a median age of 76 years (IQR: 64–83). The majority of the study population comprised patients with pneumonic infiltrates, detected by thorax CT, and most (*n* = 137, 55.9%) required oxygen supplementation at hospital admission, which corresponded to a WHO outcome classification of 4, based on the Ordinal Scale for Clinical Improvement as depicted in Supplementary Data Table S1 [27]. The median follow-up time was 108 days (IQR: 19.5–162.5 days). The numbers of deaths after 30, 60, and 90 days were 44 (19.91%), 52 (23.64%), and 52 (25.37%), respectively. The total number of deaths during follow up was 54. Taken the 60-day post-hospital mortality, 60 days after discharge 13 from 180 patients died after initially surviving COVID-19 hospitalization. Twenty-six patients with shorter follow-up than 60 days and 39 patients that already died in the hospital were excluded for this analysis.

### 3.2. Soluble ST2 Serum Content Is Significantly Associated with Worse Overall Survival

To assess a potential prognostic impact of investigated inflammatory molecules on Overall Survival, a univariable Cox-Regression model was performed. In our univariable analysis, age (measured in years) and sST2 (measured in ng/mL) are associated with worse overall survival in our study cohort (HR = 1.364, *p* = 0.001). Elevated sST2 levels remained significantly associated with higher mortality adjusting for age in the multivariable analysis (HR = 1.353, *p* = 0.002). All other investigated inflammatory markers showed no significant association with worse overall survival. Detailed results are listed in Table 4.

### 3.3. Elevated sST2 Acts as an Potential Prognostic Factor for 30-, 60- and 90-Days Mortality, Elevated 20S Proteasome Levels Were Associated with Worse 30-Days Mortality

**Figure 1:**
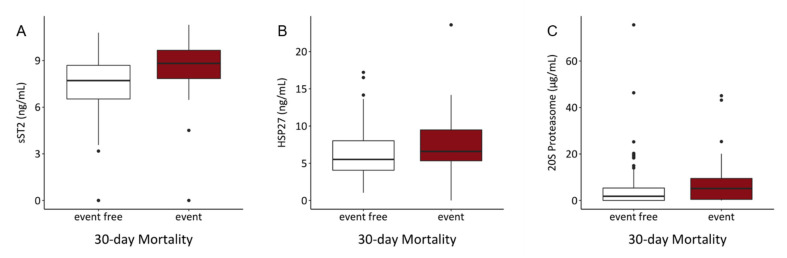
Comparison of serum contents of (**A**) sST2, (**B**) HSP27 and (**C**) 20S proteasome showed significant elevations of investigated parameters in the event group. (sST2: *p* < 0.001, HSP27: *p* = 0.04, 20S proteasome: *p* = 0.017).

To investigate the association of assessed inflammatory markers with 30-, 60- and 90-day mortality, uni- and multivariable logistic regression models were computed. Since their follow up time was shorter than 30 days, 24 patients were excluded from the analysis. Within 30 days, 44 patients died. Increased levels of serum sST2 and 20S proteasome were associated with higher 30-days mortality in a univariable analysis (sST2: OR = 1.536, *p* < 0.001; 20S proteasome: OR = 1.037, *p* = 0.004). None of the other assessed biomarkers were significantly associated with worse prognosis in 30-days mortality. In multivariable analysis with inclusion of sST2, 20S proteasome and age, only sST2 remained significantly associated with the outcome (OR = 1.457, *p* = 0.004) (Table 5). Comparing serum content of sST2, HSP27 and 20S proteasome, patients in the event group showed significantly elevated levels of these markers (Figure 1, Table 3). TNFR1 and cCK18 serum contents for all binary endpoints in event and non-event groups are depicted in Figures S1–S6.

**Figure 2:**
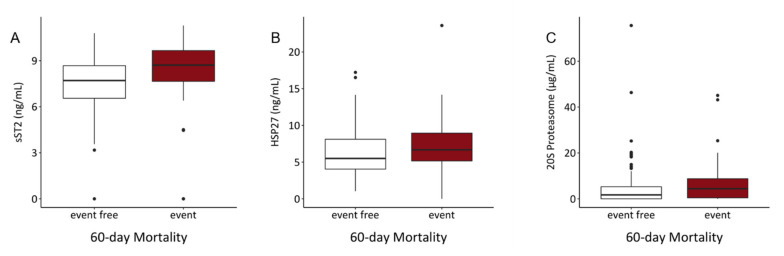
Serum levels of (**A**) sST2, (**B**) HSP27 and (**C**) 20S proteasome of patients not surviving 60 days differed significantly from patients surviving the first 60 days. (sST2: *p* < 0.001, HSP27: *p* = 0.036, 20S proteasome: *p* = 0.021).

For 60-day mortality outcome, 25 patients were excluded since their follow up time was shorter than 60 days. Only sST2 was significantly associated with increased 60-day mortality risk (univariable: OR = 1.335, *p* = 0.007; multivariable: OR = 1.298, *p* = 0.016), whereas the other markers showed no significant effect on the 60-day outcome (Table 6). Taken the median serum levels of patients who died within 60 days, they should a significant increment of sST2, HSP27 and 20S proteasome (Figure 2, Table 3).

**Figure 3:**
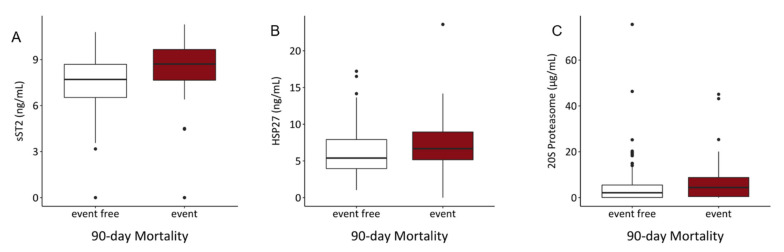
Comparison of serum contents of (**A**) sST2, (**B**) HSP27 showed significant elevations of sST2 (*p* < 0.001) and HSP27 (*p* = 0.023). In (**C)** no significant alteration of 20S proteasome levels in the event group was detected (*p* = ns).

For the analysis of 90-day mortality, 40 patients were excluded due to shorter follow-up time. In our study cohort, 52 patients died during the first 90 days. In the univariable and multivariable logistic regression model, increased values of sST2 were significantly associated with higher 90-day mortality risk in both, the univariable (OR = 1.335, *p* = 0.007) and multivariable (OR = 1.304, *p* = 0.014) analyses. (Table 7) Serum levels of sST2 and HSP27 were also elevated in non-surviving patients (Figure 3, Table 3).

### 3.4. sST2 and 20S Proteasome Serum Levels Are Significant Predictors for In-Hospital Mortality

**Figure 4:**
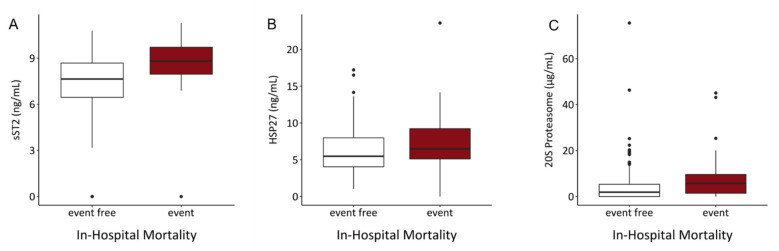
Comparison of serum contents of (**A**) sST2 and (**C**) 20S proteasome showed significant elevations in patients who died in the hospital when compared to surviving hospital patients. (sST2: *p* < 0.001, 20S proteasome: *p* = 0.001). Comparison of HSP27 serum content (**B**) showed no significant difference between these two groups (*p* = ns).

To evaluate the prognostic impact of investigated biomarkers on hospital survival, logistic regression models were performed. Of 245 included patients, 39 died in the hospital. In univariable analysis, sST2 (OR = 1.697, *p* < 0.001) and 20S proteasome (OR = 1.044, *p* = 0.019) were significantly associated with increased risk of in-hospital mortality. This effects also remained in multivariable analysis (OR = 1.631 and 1.041, *p* < 0.001 and 0.033, respectively). The other investigated parameters showed no significant prognostic impact on in-hospital mortality (Table 8). sST2 and 20S proteasome levels were significantly elevated in patients who died during hospitalization (Figure 4, Table 3).

### 3.5. Serum Content of sST2, HSP27, and 20S Proteasome Might Predict Risk of Intubation and Requirement of Oxygen Therapy

We further evaluated the impact of inflammatory markers on endpoints such as the requirement of invasive ventilation or oxygen therapy. In our study cohort, 43 patients required invasive ventilation during hospitalization and oxygen therapy at hospital admission was needed in 179 cases. HSP27, sST2 and 20S proteasome serum levels were elevated in patients requiring invasive ventilation or oxygen therapy.

**Figure 5:**
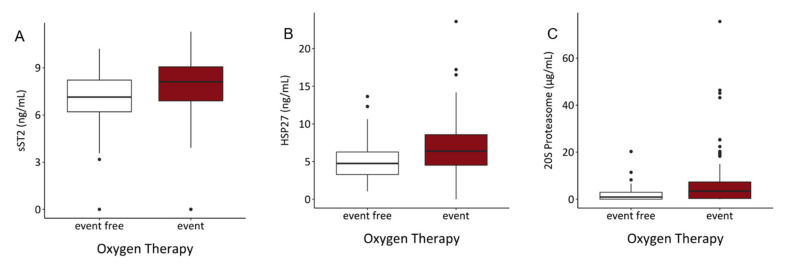
Serum levels of (**A**) sST2, (**B**) HSP27 and (**C**) 20S proteasome showed significant elevations of investigated parameters in the event group. (sST2: *p* < 0.001, HSP27: *p* < 0.001, 20S proteasome: *p* < 0.001).

HSP27 (OR = 1.218, *p* < 0.001), sST2 (OR = 1.258, *p* = 0.003) and 20S proteasome (OR = 1.177, *p* < 0.001) were significantly associated with the requirement of oxygen therapy at hospital admission in the univariable analysis. Increased values of these molecules were also significantly associated with the need for oxygen therapy in the multivariable analysis (OR = 1.134, 1.175 and 1.147, *p* = 0.037, 0.046 and 0.004, respectively) (Table 9). Serum levels of these inflammatory molecules were also significantly increased in patients requiring oxygen therapy at hospital admission (Figure 5).

**Figure 6:**
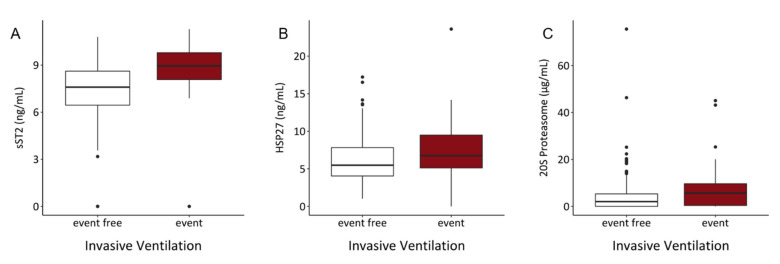
Serum levels of (**A**) sST2, (**B**) HSP27 and (**C**) 20S proteasome showed significant elevations of investigated parameters in the event group. (sST2: *p* < 0.001, HSP27: *p* = 0.034, 20S proteasome: *p* = 0.016).

In the univariable analysis HSP27 (OR = 1.108, *p* = 0.041), sST2 (OR = 1.79, *p* < 0.001) and 20S proteasome (OR = 1.039, *p* = 0.031) were identified as significant predictors for the risk of intubation. In the multivariable analysis, only sST2 (OR = 1.709, *p* < 0.001) remained significantly associated with the outcome (Table 10). Comparing the median concentrations of sST2, HSP27 and 20S proteasome, patients requiring invasive ventilations showed significant increment of these inflammatory molecules (Figure 6).

### 3.6. Correlations between sST2 with 20S Proteasome, HSP27, and TNFR1

**Figure 7:**
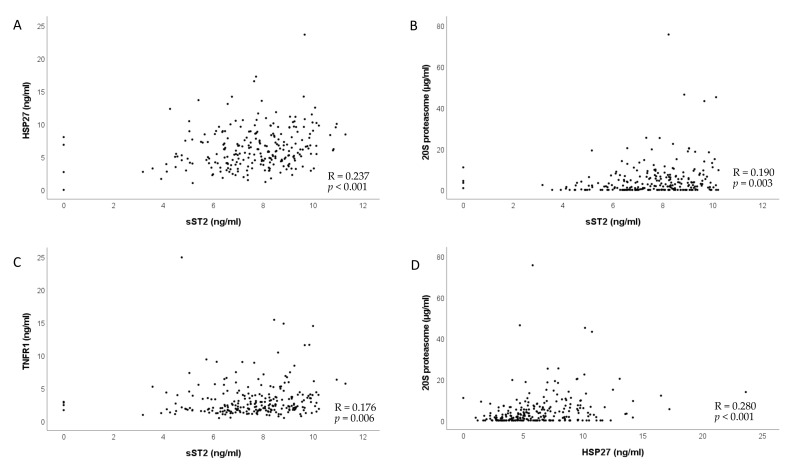
Serum levels of (**A**) sST2 and HSP27, (**B**) sST2 and 20S proteasome, (**C**) sST2 and TNFR1 and (**D**) HSP27 and 20S proteasome showed significant correlations using Spearman’s rank correlation.

**Figure 8:**
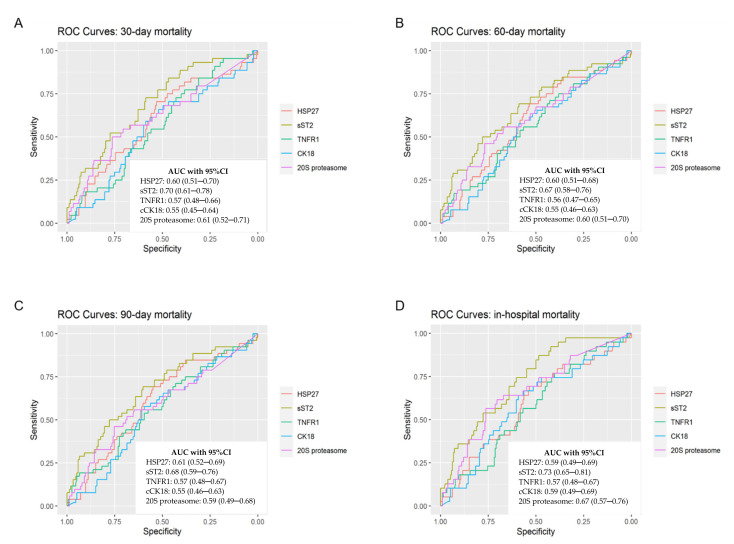
ROCs for binary endpoints were computed and AUC with 95% CI were analyzed to determine diagnostic accuracy. This figure shows ROCs for (**A**) 30-day mortality, (**B**) 60-day mortality, (**C**) 90-day mortality and (**D**) in-hospital mortality.

To identify potential correlations between the investigated biomarkers, we performed the Spearman rank correlation analyses. We found that ST2 was positively correlated with 20S proteasome, HSP27, and TNFR1 and that HSP27 was positively correlated with 20S proteasome (Figure 7). ROC curves and Area under the curve (AUC) are depicted in Figure 8.

## 4. Discussion

In this study, we examined the presence of soluble HSP27, sST2, TNFR1, cCK18, and 20S proteasome in serum samples obtained at hospital admission from patients with COVID-19. We confirmed previous studies which showed elevated sST2 levels in severe and fatal COVID-19 cases [25,28,29]. We also identified sST2 as a potential prognostic marker of overall mortality, 30-,60- and 90-day mortality, and in-hospital mortality. Serum sST2, 20S proteasome and HSP27 were also associated with the need of oxygen therapy or invasive ventilation. To our knowledge, this study was the first to examine HSP27 and 20S proteasome concentrations in SARS-CoV-2 positive patients. Moreover, we showed that elevated serum sST2 and 20S proteasome levels at hospital admission were significantly associated with increased risk of in-hospital mortality in uni- and multivariable logistic regression.

### 4.1. sST2

ST2 is a member of the IL-1 receptor family. It is expressed in two main isoforms: a transmembrane cellular (ST2L) and a soluble (sST2) form. ST2 is the receptor for IL-33, which is secreted by living cells in response to cell damage [30]. Alveolar epithelial cells in the lung and cardiac myocytes were previously identified as main sources of sST2 [22]. Because sST2 is inducible by inflammatory stimuli, it is elevated in a large number of diseases, including sepsis, trauma, burns, and heart failure [21,25,31,32,33]. Another study assessed serum sST2 levels in patients with chronic heart failure [31] and showed that the median sST2 concentration was 3.631ng/mL. In contrast, serum sST2 levels were greater than 400 ng/mL in patients on day 1 after a left ventricular assist device (LVAD) implantation, and subsequently, levels normalized by the end of the first postoperative week [34]. In comparison, the median sST2 value in our study cohort was 7.853 ng/mL (IQR: 6.584–8.935). Therefore, the median serum sST2 levels in patients with COVID-19 were twice as high as the levels found in patients with chronic heart failure. Hacker et al. [32] and Brunner et al. [21] detected a median serum level of 99–316 pg/mL in healthy controls. This finding suggested that patients with COVID-19 infections had undergone a remarkable production of inflammatory molecules by the time they were admitted to the hospital. We also found a correlation of patients suffering from heart failure and worse outcome in all endpoints investigated, which may be re related to a two-hit hypothesis: already elevated levels of sST, such as in chronic heart failure patients [31] plus the inflammatory secretion during COVID-19 as seen in our results lead to massive increment of sST2-mediated pathways resulting in worse outcome. This is underlined by the fact that we found sST2 as a potential prognostic factor for in-hospital mortality, 30-, 60- and 90-day mortality. This finding supported the hypothesis that patients with severe COVID-19 exhibited strong inflammatory activation [5,6,8], and it confirmed previous findings from Abers et al. [25] that identified sST2 as a marker that clearly distinguished between patients with increased risk and patients with low risk of mortality.

### 4.2. HSP27

HSP27 acts as an intracellular chaperone to maintain normal cell functions, but it is also active extracellularly. HSPs are involved in apoptosis inhibition, cytoprotection, and immunomodulation [35,36,37,38]. Moreover, HSP27 may play a major role in platelet aggregation by modifying actin polymerization [38,39]. Tian et al. [39] found an association between a high thrombus burden and elevated HSP27 levels in patients with ST elevation myocardial infarctions. In addition, recently, HSP27 was identified as prognostic marker in inflammatory diseases, such as COPD and trauma-related ARDS [23,24,37,40,41]. Therefore, we hypothesized that, in patients with COVID-19, elevated HSP27 might be associated with lung injury. Furthermore, the increase in HSP27 might have been related to thromboinflammation, which is the coordinated activation of inflammatory and thrombotic responses [38]. Indeed, this condition might explain some of the key aspects of morbidity and mortality observed in patients with COVID-19 [42].

HSP27 serum levels were previously assessed in healthy volunteers, who exhibited a median concentration of 1.482 ng/mL [43]. In our study cohort, the median HSP27 level at hospital admission was 5.839 ng/mL (IQR: 4.070–8.316), which was about four-fold higher than the levels found in healthy controls. In our study cohort, HSP27 was significantly associated with increased risk for invasive ventilation or the need of oxygen therapy, which could indicate an extensive damage of the lung in these patients.

### 4.3. 20S Proteasome

Roth et al. [19] previously described elevated core 20S proteasome levels in critically ill patients, particularly those with sepsis. Core 20S proteasome is a marker of increased apoptotic turnover. Therefore, we investigated whether 20S proteasome levels could act as a prognostic marker for overall and in-hospital mortality. Surprisingly, we found that increased 20S proteasome levels were not significantly associated with overall-, 30-,60- and 90-day mortality, but we found that 20S proteasome was a significant predictor for the risk of invasive ventilation or the need of oxygen therapy. Increased 20S serum proteasome was also significantly associated with higher in-hospital mortality risk. Elevated 20S proteasome was found in several other pathological conditions such as carcinoma [44], burn injuries [45] and autoimmune diseases [46] Lately, the therapeutic potential of proteasome inhibitors have been discussed and several studies showed increased 20S proteasome levels in the alveolar space during ARDS in an active configuration [47,48,49,50]. In our patient cohort the median serum level of 20S proteasome was 2.2 µg/mL whereas serum levels of healthy controls were assessed 1.1 ± 0.26 µg/mL in a study from Hetz et al. [51]. These observations confirm our findings that patients in our study cohort with elevated 20S proteasome levels suffer from severe tissue damage and hyper-activated immune system, whereas these factors together lead to increased apoptosis and thus probably higher risk of invasive ventilation or higher in-hospital mortality [52].

### 4.4. Survival

Our population comprised patients primarily hospitalized in a non-intensive care unit (ICU) setting. Patients with severe diseases, up to WHO scale 5 (high-flow oxygen therapy), could be treated outside the ICU. Patients that required non-invasive or invasive ventilation had to be transferred to the ICU. Our cohort had a mortality of 19.91% after 30 days. However, the mortality of our study cohort increased to 23.64% after 90 days, respectively. Additionally, the fact that after discharge from the hospital, nearly 8% of patients died after initially surviving COVID-19, reflect the possibly longer-term effects of the SARS-CoV-2 infection.

### 4.5. Limitations

The main limitation of this study was the lack of a matched reference group without a COVID-19 history, for survival estimations in this vulnerable group of older patients (median age 76 years) and the performance in a single-center setting. However, our survival results suggested that COVID-19 also had an impact on survival after successful treatment of the acute infection and clearance of the virological disease. Also, the lack of patients receiving ECMO in our study population due to transfer to another hospital may underestimate the prognostic value for severe or critical disease of our parameters investigated.

Additionally, as a result of its retrospective character, laboratory parameters such as d-dimer, fibrinogen, lymphocyte and leukocyte count at hospital admission were not available for all patients and could therefore not be used for our analysis. Although our parameters investigated don’t have sufficient accuracy to be used in clinics as such the association of sST2, HSP27 and 20S proteasome in combination with the previously mentioned laboratory parameters with disease outcome may be a great avenue for further investigations. The study of Janik et al. [53], where they showed a prognostic and diagnostic impact of fibrinogen, neutrophil-to-lymphocyte ratio (NLR), and platelet-to-lymphocyte (PLR) ratio on thymic epithelial tumors outcome could act as an example for further COVID-19 biomarker investigations.

## 5. Conclusions

With this study, we evidenced that a high level of sST2 could serve as prognostic marker for disease severity and overall survival. We demonstrated that serum sST2 was an early prognostic marker for overall mortality, 30-, 60-, and 90-day mortality and in-hospital mortality, and the need for invasive ventilation or oxygen therapy. Moreover, we showed that besides sST2, also 20S proteasome, and HSP27 were significantly associated with a subsequent requirement for invasive ventilation during hospitalization. In addition, we evidenced that the augmented release of these inflammatory molecules was associated with the need for oxygen therapy at hospital admission. We also detected a significant increment in 20S proteasome and sST2 levels in patients that died during the hospital stay. Even if the parameters don’t have sufficient accuracy to be used in clinics as such, our analysis provided a fundament for further investigations such as combination of sST2, HSP27, and 20S proteasome with commonly measured laboratory parameters such as fibrinogen and d-dimer. Our findings also contributed to a broader understanding of the systemic inflammation response in patients with COVID-19 and might help identify high risk patients at admission.

## Figures and Tables

**Table 1:** Demographic characteristics for the study population at hospital admission.

		30-Day Mortality			60-Day Mortality			90-Day Mortality			In-Hospital Mortality		
Characteristics	All Patients (*n* = 245)	Event (*n* = 44)	Event-Free (*n* = 177)	*p*-Value	Event (*n* = 52)	Event-Free (*n* = 168)	*p*-Value	Event (*n* = 52)	Event-Free (*n* = 153)	*p*-Value	Event (*n* = 39)	Event-Free (*n* = 206)	*p*-Value
Age (years)	76 (64–83)	80.5 (74–86)	74 (62–83)		80 (74–86)	74 (62–83)		80 (74–86)	74 (62–83)		81 (74–86)	74 (62.25–82)	
Sex													
Female	113 (46.12%)	16 (36.36%)	83 (46.89%)	ns	19 (36.54%)	80 (47.62%)	ns	19 (36.54%)	69 (45.1%)	ns	16 (41.03%)	97 (47.09%)	ns
Male	132 (53.88%)	28 (63.64%)	94 (53.11%)	ns	33 (63.46%)	88 (52.38%)	ns	33 (63.46%)	84 (54.9%)	ns	23 (58.97%)	109 (52.91%)	ns
Comorbidities													
Diabetes	98 (40.2%)	19 (43.2%)	72 (40.9%)	ns	25 (48.1%)	66 (39.5%)	ns	25 (48.1%)	60 (39.5%)	ns	18 (46.2%)	67 (40.6%)	ns
Hypertension	183 (74.4%)	35 (79.5%)	131 (74.4%)	ns	43 (82.7%)	122 (73.1%)	ns	43 (82.7%)	111 (73.0%)	ns	33 (84.6%)	121 (73.3%)	ns
Transplantation	2 (0.8%)	0 (0%)	1 (0.6%)	ns	0 (0%)	1 (0.6%)	ns	0 (0.0%)	1 (0.7%)	ns	0 (0.0%)	1 (0.6%)	ns
Liver cirrhosis	2 (0.8%)	1 (2.4%)	1 (0.6%)	ns	1 (2.0%)	1 (0.6%)	ns	1 (2.0%)	1 (0.7%)	ns	1 (2.7%)	1 (0.6%)	ns
Heart failure	64 (26.1%)	21 (48.8%)	38 (21.5%)	<0.01	22 (43.1%)	37 (22.0%)	<0.01	22 (43.1%)	36 (23.5%)	0.01	18 (47.4%)	40 (24.1%)	<0.01
Medication at admission													
ACE Inhibitors	75 (30.6%)	15 (34.1%)	52 (29.5%)	ns	18 (34.6%)	49 (29.3%)	ns	18 (34.6%)	44 (28.9%)	ns	12 (30.8%)	50 (30.3%)	ns
Angiotensin-Receptor Blocker	87 (35.5%)	11 (25.0%)	69 (39.2%)	ns	15 (28.8%)	64 (38.3%)	ns	15 (28.8%)	60 (39.5%)	ns	10 (25.6%)	65 (39.4%)	ns
Calcium Inhibitors	32 (13.1%)	6 (13.6%)	20 (11.4%)	ns	6 (11.5%)	20 (12.0%)	ns	6 (11.5%)	20 (13.2%)	ns	6 (15.4%)	20 (12.2%)	ns
Glucocorticoids	14 (5.7%)	5 (11.4%)	7 (4.0%)	ns	5 (9.6%)	7 (4.2%)	ns	5 (9.6%)	7 (4.6%)	ns	3 (7.7%)	9 (5.5%)	ns
Aspirin	52 (21.2%)	8 (18.2%)	41 (23.3%)	ns	12 (23.1%)	38 (22.2%)	ns	12 (23.1%)	35 (23.0%)	ns	7 (17.9%)	40 (24.2%)	ns
Heparin	5 (2.0%)	2 (4.5%)	3 (1.7%)	ns	2 (3.8%)	3 (1.8%)	ns	2 (3.8%)	3 (2.0%)	ns	1 (2.6%)	4 (2.4%)	ns
NOACs	56 (22.9%)	15 (34.1%)	36 (20.5%)	ns	15 (28.8%)	36 (21.6%)	ns	15 (28.8%)	34 (22.4%)	ns	11 (28.2%)	38 (23.0%)	ns
Hospitalization (days)	10 (6–16)												

Values are the median (interquartile range) or the number (%), as indicated. Percentages refer to event or no-event group; ACE, angiotensin converting enzyme; NOAC, new oral anticoagulants; ns, non-significant.

**Table 2:** WHO outcome classification codes for the study population.

		30-Day Mortality		60-Day Mortality		90-Day Mortality		In-Hospital Mortality	
Characteristics	All Patients (*n* = 245)	Event (*n* = 44)	Event-Free (*n* = 177)	Event (*n* = 52)	Event-Free (*n* = 168)	Event (*n* = 52)	Event-Free (*n* = 153)	Event (*n* = 39)	Event-Free (*n* = 206)
WHO Outcome Classification at admission									
2	3 (1.22%)	0 (0%)	3 (1.69%)	0 (0%)	3 (1.79%)	0 (0%)	2 (1.31%)	0 (0%)	3 (1.46%)
3	63 (25.71%)	8 (18.18%)	50 (28.25%)	11 (21.15%)	47 (27.98%)	11 (21.15%)	43 (28.1%)	7 (17.95%)	56 (27.18%)
4	137 (55.92%)	22 (50%)	100 (56.5%)	26 (50%)	96 (57.14%)	26 (50%)	86 (56.21%)	18 (46.15%)	119 (57.77%)
5	41 (16.73%)	13 (29.55%)	24 (13.56%)	14 (26.92%)	22 (13.1%)	14 (26.92%)	22 (14.38%)	13 (33.33%)	28 (13.59%)
6	1 (0.41%)	1 (2.27%)	0 (0%)	1 (1.92%)	0 (0%)	1 (1.92%)	0 (0%)	1 (2.56%)	0 (0%)
at discharge									
0	26 (10.61%)	0 (0%)	19 (10.73%)	0 (0%)	19 (11.31%)	0 (0%)	19 (12.42%)	0 (0%)	26 (12.62%)
1	14 (5.71%)	2 (4.55%)	11 (6.21%)	3 (5.77%)	10 (5.95%)	3 (5.77%)	9 (5.88%)	1 (2.56%)	13 (6.31%)
2	131 (53.47%)	2 (4.55%)	115 (64.97%)	5 (9.62%)	111 (66.07%)	5 (9.62%)	105 (68.63%)	0 (0%)	131 (63.59%)
3	18 (7.35%)	0 (0%)	17 (9.6%)	0 (0%)	17 (10.12%)	0 (0%)	13 (8.5%)	0 (0%)	18 (8.74%)
4	12 (4.9%)	1 (2.27%)	10 (5.65%)	1 (1.92%)	10 (5.95%)	1 (1.92%)	7 (4.58%)	1 (2.56%)	11 (5.34%)
5	1 (0.41%)	0 (0%)	1 (0.56%)	1 (1.92%)	0 (0%)	1 (1.92%)	0 (0%)	0 (0%)	1 (0.49%)
7	1 (0.41%)	0 (0%)	1 (0.56%)	0 (0%)	1 (0.6%)	0 (0%)	0 (0%)	0 (0%)	1 (0.49%)
8	42 (17.14%)	39 (88.64%)	3 (1.69%)	42 (80.77%)	0 (0%)	42 (80.77%)	0 (0%)	37 (94.87%)	5 (2.43%)

Values are the median (interquartile range) or the number (%), as indicated. Percentages refer to event or no-event group; WHO, World Health Organization.

**Table 3:** Median serum content of assessed molecules at hospital admission for endpoints investigated.

		30-Day Mortality		60-Day Mortality		90-Day Mortality		In-Hospital Mortality	
Molecules	All Patients (*n* = 245)	Event (*n* = 44)	Event-Free(*n* = 177)	Event (*n* = 52)	Event-Free (*n* = 168)	Event (*n* = 52)	Event-Free (*n* = 153)	Event (*n* = 39)	Event-Free (*n* = 206)
sST2 (ng/mL)	7.9	8.8 (7.8–9.7)	7.7 (6.5–8.7)	8.7 (7.6–9.6)	7.7 (6.5–8.7)	8.7 (7.6–9.7)	7.7 (6.5–8.7)	8.8 (7.9–9.8)	7.7 (6.5–8.7)
HSP27 (ng/mL)	5.8	6.6 (5.3–9.5)	5.5 (4.1–8.0)	6.7 (5.1–9.0)	5.5 (4.0–8.2)	6.7 (5.0–9.0)	5.4 (4.0–8.1)	6.5 (5.0–9.5)	5.5 (4.0–8.1)
20S proteasome (µg/mL)	2.2	5.2 (0.3–9.6)	1.8 (0–5.4)	4.4 (0.3–9.2)	5.3 (0–5.3)	4.4 (0.3–9.2)	2.1 (0–5.6)	5.7 (1.1–9.8)	2.0 (0–5.4)
cCK18 (U/L)	162	151 (127–233)	173 (130–274)	151 (127–234)	170 (130–259)	151 (127–234)	170 (130–268)	148 (126–233)	171 (131–265)
TNFR1 (ng/mL)	2.4	2.7 (2.0–3.9)	2.3 (1.7–3.9)	2.7 (1.9–4.0)	2.4 (1.7–3.9)	2.7 (1.9–4.0)	2.3 (1.7–3.7)	2.6 (2.0–3.6)	2.3 (1.7–3.9)

Data are given as median with interquartile range; sST2, soluble suppression of tumorigenicity 2; HSP27, heat shock protein 27; cCK18, caspase cleaved cytokeratin 18; TNFR1, tumor necrosis factor receptor 1.

**Table 4:** Univariable and multivariable Cox-Regression in association with overall survival (*n* = 245).

Prognostic Markers	Hazard Ratio	Lower CL	Upper CL	*p*-Value
Univariable Cox-Regression				
Age (years)	1.047	1.02	1.074	<0.001
HSP27 (ng/mL)	1.083	0.998	1.176	0.055
sST2 (ng/mL)	1.364	1.13	1.647	0.001
TNFR1 (ng/mL)	1.053	0.98	1.132	0.162
cCK18 (1000 U/L)	0.595	0.13	2.735	0.505
20S proteasome (µg/mL)	1.019	0.998	1.041	0.076
Multivariable Cox-Regression				
Age (years)	1.047	1.019	1.075	<0.001
sST2 (ng/mL)	1.353	1.116	1.639	0.002

CL, confidence-limit; sST2, soluble suppression of tumorigenicity 2; HSP27, heat shock protein 27; cCK18, caspase cleaved cytokeratin 18; TNFR1, tumor necrosis factor receptor 1.

**Table 5:** Univariable and multivariable logistic regression in association with 30-day mortality (*n* = 221).

Prognostic Markers	Odds Ratio	Lower CL	Upper CL	*p*-Value
Univariable Logistic Regression				
Age (years)	1.056	1.023	1.094	0.001
HSP27 (ng/mL)	1.102	0.997	1.22	0.056
sST2 (ng/mL)	1.536	1.216	1.991	<0.001
TNFR1 (ng/mL)	1.046	0.931	1.16	0.408
cCK18 (1000 U/L)	0.723	0.101	3.097	0.702
20S proteasome (µg/mL)	1.037	1.002	1.077	0.044
Multivariable Logistic Regression				
Age (years)	1.059	1.024	1.1	0.002
sST2 (ng/mL)	1.457	1.144	1.911	0.004
20S proteasome (µg/mL)	1.034	0.997	1.076	0.072

CL, confidence-limit; sST2, soluble suppression of tumorigenicity 2; HSP27, heat shock protein 27; cCK18, caspase cleaved cytokeratin 18; TNFR1, tumor necrosis factor receptor 1.

**Table 6:** Univariable and multivariable logistic regression in association with 60-day mortality (*n* = 220).

Prognostic Markers	Odds Ratio	Lower CL	Upper CL	*p*-Value
Univariable Logistic Regression				
Age (years)	1.052	1.023	1.087	<0.001
HSP27 (ng/mL)	1.092	0.992	1.204	0.071
sST2 (ng/mL)	1.332	1.091	1.659	0.007
TNFR1 (ng/mL)	1.07	0.964	1.186	0.19
cCK18 (1000 U/L)	0.566	0.081	2.42	0.502
20S proteasome (µg/mL)	1.032	0.997	1.07	0.076
Multivariable Logistic Regression				
Age (years)	1.051	1.02	1.086	0.002
sST2 (ng/mL)	1.298	1.061	1.624	0.016

CL, confidence-limit; sST2, soluble suppression of tumorigenicity 2; HSP27, heat shock protein 27; cCK18, caspase cleaved cytokeratin 18; TNFR1, tumor necrosis factor receptor 1.

**Table 7:** Univariable and multivariable logistic regression in association with 90-day mortality (*n* = 205).

Prognostic Markers	Odds Ratio	Lower CL	Upper CL	*p*-Value
Univariable Logistic Regression				
Age (years)	1.053	1.022	1.088	0.001
HSP27 (ng/mL)	1.096	0.996	1.209	0.061
sST2 (ng/mL)	1.335	1.095	1.663	0.007
TNFR1(ng/mL)	1.098	0.984	1.236	0.098
cCK18 (1000 U/L)	0.536	0.076	2.296	0.463
20S proteasome (µg/mL)	1.027	0.993	1.065	0.122
Multivariable Logistic Regression				
Age (years)	1.051	1.02	1.087	0.002
sST2 (ng/mL)	1.304	1.068	1.631	0.014

CL, confidence-limit; sST2, soluble suppression of tumorigenicity 2; HSP27, heat shock protein 27; cCK18, caspase cleaved cytokeratin 18; TNFR1, tumor necrosis factor receptor 1.

**Table 8:** Univariable and multivariable logistic regression in association with in-hospital mortality (*n* = 245).

Prognostic Markers	Odds Ratio	Lower CL	Upper CL	*p*-Value
Univariable Logistic Regression				
Age (years)	1.062	1.027	1.104	<0.001
HSP27 (ng/mL)	1.101	0.993	1.22	0.063
sST2 (ng/mL)	1.697	1.316	2.253	<0.001
TNFR1 (ng/mL)	1.079	0.963	1.2	0.16
cCK18 (1000 U/L)	0.291	0.019	1.957	0.298
20S proteasome (µg/mL)	1.044	1.008	1.085	0.019
Multivariable Logistic Regression				
Age (years)	1.068	1.029	1.114	0.001
sST2 (ng/mL)	1.631	1.245	2.21	<0.001
20S proteasome (µg/mL)	1.041	1.003	1.085	0.033

CL, confidence-limit; sST2, soluble suppression of tumorigenicity 2; HSP27, heat shock protein 27; cCK18, caspase cleaved cytokeratin 18; TNFR1, tumor necrosis factor receptor 1.

**Table 9:** Univariable and multivariable logistic regression in association with requirement of oxygen therapy (*n* = 245).

Prognostic Markers	Odds Ratio	Lower CL	Upper CL	*p*-Value
Univariable Logistic Regression				
Age (years)	0.995	0.974	1.016	0.662
HSP27 (ng/mL)	1.218	1.091	1.374	<0.001
sST2 (ng/mL)	1.258	1.082	1.475	0.003
TNFR1 (ng/mL)	1.107	0.98	1.289	0.146
cCK18 (1000 U/L)	0.747	0.213	2.985	0.651
20S proteasome (µg/mL)	1.177	1.084	1.3	<0.001
Multivariable Logistic Regression				
HSP27 (ng/mL)	1.134	1.012	1.282	0.037
sST2 (ng/mL)	1.175	1.003	1.38	0.046
20S proteasome (µg/mL)	1.147	1.054	1.269	0.004

CL, confidence-limit; sST2, soluble suppression of tumorigenicity 2; HSP27, heat shock protein 27; cCK18, caspase cleaved cytokeratin 18; TNFR1, tumor necrosis factor receptor 1.

**Table 10:** Univariable and multivariable logistic regression in association with requirement of invasive ventilation (*n* = 245).

Prognostic Markers	Odds Ratio	Lower CL	Upper CL	*p*-Value
Univariable Logistic Regression				
Age (years)	1.053	1.021	1.09	0.002
HSP27 (ng/mL)	1.108	1.003	1.225	0.041
sST2 (ng/mL)	1.79	1.39	2.373	<0.001
TNFR1 (ng/mL)	1.065	0.951	1.182	0.24
cCK18 (1000 U/L)	0.806	0.112	3.45	0.799
20S proteasome (µg/mL)	1.039	1.004	1.079	0.031
Multivariable Logistic Regression				
Age (years)	1.055	1.02	1.096	0.003
HSP27 (ng/mL)	1.028	0.915	1.155	0.635
sST2 (ng/mL)	1.709	1.306	2.31	<0.001
20S proteasome (µg/mL)	1.032	0.993	1.074	0.094

CL, confidence-limit; sST2, soluble suppression of tumorigenicity 2; HSP27, heat shock protein 27; cCK18, caspase cleaved cytokeratin 18; TNFR1, tumor necrosis factor receptor 1.

## Data Availability

All data and materials support our published claims and comply with field standards. All data are available upon request.

## Supplementary Materials

The following are available online at https://www.mdpi.com/article/10.3390/biology10111186/s1, Figure S1: Serum content of CK18 and TNFR1 revealed no significant difference between the event and event-free group. CK18, cytokeratin 18; TNFR1, tumor necrosis factor receptor 1, Figure S2: Serum content of CK18 and TNFR1 revealed no significant difference between the event and event-free group. CK18, cytokeratin 18; TNFR1, tumor necrosis factor receptor 1, Figure S3: Serum content of CK18 and TNFR1 revealed no significant difference between the event and event-free group. CK18, cytokeratin 18; TNFR1, tumor necrosis factor receptor 1, Figure S4: Serum content of CK18 and TNFR1 revealed no significant difference between the event and event-free group. CK18, cytokeratin 18; TNFR1, tumor necrosis factor receptor 1, Figure S5: Serum content of CK18 and TNFR1 revealed no significant difference between the event and event-free group. CK18, cytokeratin 18; TNFR1, tumor necrosis factor receptor 1, Figure S6: Serum content of CK18 and TNFR1 revealed no significant difference between the event and event-free group. CK18, cytokeratin 18; TNFR1, tumor necrosis factor receptor 1, Table S1: WHO-Outcome Classification.
