# Supplementary material for: Clinical Relevance of Elevated Soluble ST2, HSP27 and 20S Proteasome at Hospital Admission in Patients with COVID-19
Source: Biology (Basel). 2021 Nov 15;10(11):1186. doi: 10.3390/biology10111186 (PMC8615143; doi:10.3390/biology10111186)
Supplement: Supplementary file 1 [file biology-10-01186-s001.zip › biology-1444885-supplementary.pdf]

biology

MDPI

Supplementary Materials: Clinical Relevance of Elevated Soluble ST2, HSP27 and 20S Proteasome at Hospital Admission in Patients with COVID-19

Ralph Wendt, Marie-Therese Lingitz, Maria Laggner [2], Michael Mildner, Denise Traxler, Alexandra Graf, Pavla Krotka, Bernhard Moser, Konrad Hoetzenecker, Sven Kalbitz, Christoph Lübbert, Joachim Beige [1,9] and Hendrik Jan Ankersmit

Table S1. WHO-Outcome Classification

Patient State	Descriptor	Score
Uninfected	No clinical or virological evidence of infection	0
Ambulatory	No limitation of activities	1
	Limitation of activities	2
Hospitalized Mild disease	Hospitalized, no oxygen therapy	3
	Oxygen by mask or nasal prongs	4
Hospitalized Severe Disease	Non-invasive ventilation or high-flow oxygen	5
	Intubation and mechanical ventilation	6
	Ventilation + additional organ support- pressors, RRT, ECMO	7
Dead[a]	Death	8

WHO-Outcome Classification RRT, renal replacement therapy; ECMO, extracorporeal membrane oxygenation [1];

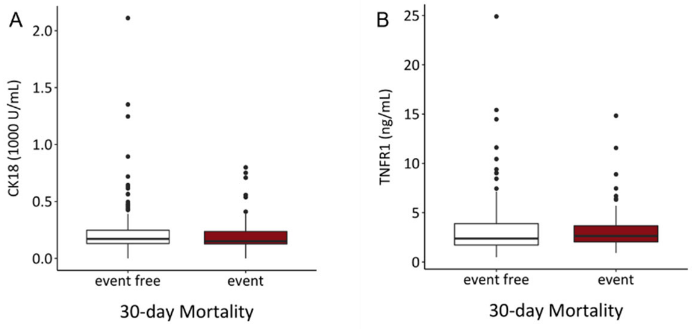

Figure S1. Serum content of CK18 and TNFR1 revealed no significant difference between the event and event-free group. CK18, cytokeratin 18; TNFR1, tumor necrosis factor receptor 1.

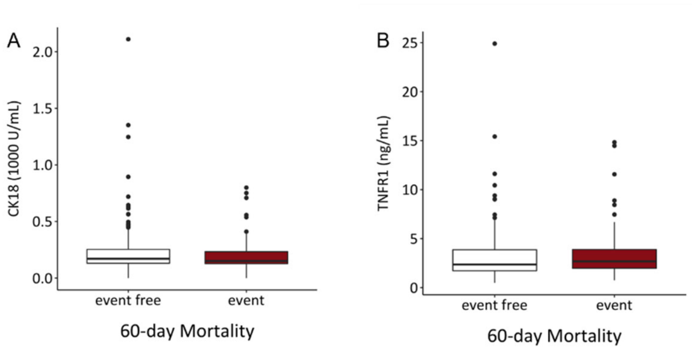

Figure S2. Serum content of CK18 and TNFR1 revealed no significant difference between the event and event-free group. CK18, cytokeratin 18; TNFR1, tumor necrosis factor receptor 1.

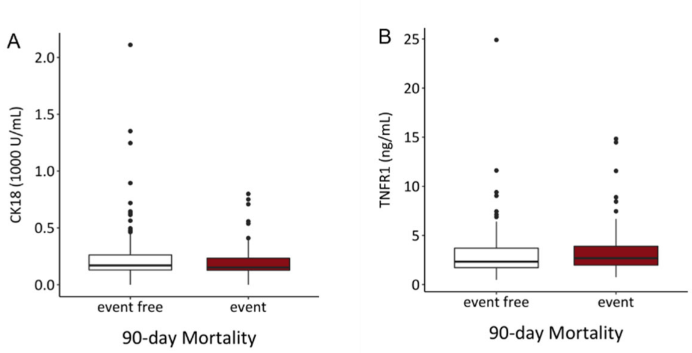

Figure S3. Serum content of CK18 and TNFR1 revealed no significant difference between the event and event-free group. CK18, cytokeratin 18; TNFR1, tumor necrosis factor receptor 1.

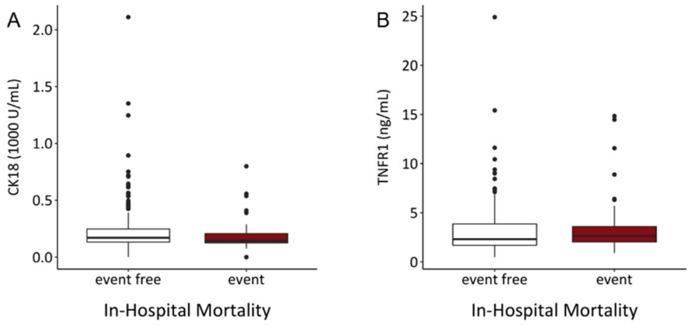

Figure S4. Serum content of CK18 and TNFR1 revealed no significant difference between the event and event-free group. CK18, cytokeratin 18; TNFR1, tumor necrosis factor receptor 1.

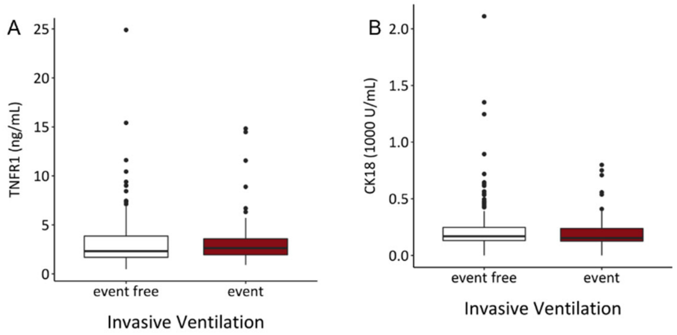

Figure S5. Serum content of CK18 and TNFR1 revealed no significant difference between the event and event-free group. CK18, cytokeratin 18; TNFR1, tumor necrosis factor receptor 1.

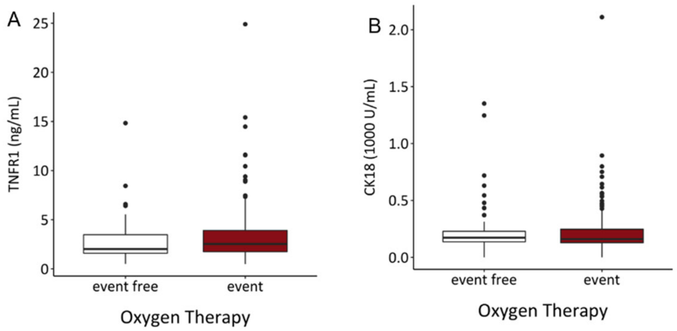

Figure S6. Serum content of CK18 and TNFR1 revealed no significant difference between the event and event-free group. CK18, cytokeratin 18; TNFR1, tumor necrosis factor receptor 1.
